# Integrated transcriptomic and metabolomic analysis suggests a potential regulatory network underlying leaf color transition in Ulmus pumila ‘Jinye’

**DOI:** 10.1186/s12870-026-09202-5

**Published:** 2026-06-08

**Authors:** Yanan Jing, Yaoqian Yang, Yamei Hu, Fuxia Bo, Songhua Dai, Shufang Yan, Shuxiang Feng, Guojun Zhang

**Affiliations:** 1 College of Horticultural Science & Technology, Hebei Normal University of Science & Technology, Qinhuangdao, China; 2 Hebei Key Laboratory of Horticultural Germplasm Excavation and Innovative Utilization, Qinhuangdao, China; 3 Hebei Higher Institute Application Technology Research and Development Center of Horticultural Plant Biological Breeding, Qinhuangdao, China; 4 Hebei Academy of Forestry and Grassland Sciences, Shijiazhuang, China

**Keywords:** *Ulmus pumila* ‘Jinye’, Leaf color transition, Pigment metabolism, Multi-omics analysis, Phytohormones

## Abstract

**Background:**

*Ulmus pumila* ‘Jinye’ is valued for its bright golden leaves, but this coloration is unstable during development, leading to a “regreening” phenomenon that reduces its ornamental value. Understanding the molecular mechanism underlying this transition is essential for stabilizing the golden leaf phenotype. This study investigates the physiological, structural, and molecular changes accompanying regreening and tests whether the Golden stage represents a state of hormone-mediated developmental arrest.

**Results:**

The regreening process was characterized across three stages: Golden, Yellowish-Green (YG), and Green. The color transition from golden to green was attributed to a marked increase in the chlorophyll-to-carotenoid ratio, resulting from net chlorophyll accumulation against a stable carotenoid background. This metabolic change was accompanied by extensive reorganization of leaf anatomy and chloroplast ultrastructure. Golden stage chloroplasts exhibited loosely stacked thylakoids, indicating delayed development, while YG and Green stages showed progressive structural maturation alongside features of early senescence, including indistinct thylakoid membranes and degenerating mitochondrial cristae. Transcriptomic and metabolomic analyses revealed coordinated reprogramming of pigment metabolism. Chlorophyll biosynthesis genes (*HEMA*, *CHLH*, *CAO*) were upregulated while degradation genes (*NYC-like*) were downregulated, driving chlorophyll accumulation. Concurrently, carotenoid biosynthesis genes, including the MEP pathway gene *DXS* and core biosynthetic gene *PSY3*, were suppressed, while cleavage genes (*CCDs*) were induced, leading to reduced carotenoid accumulation. Metabolomic data showed no accumulation of chlorophyll intermediates, suggesting a delay between transcriptional activation and functional enzyme assembly. Endogenous hormone quantification showed that multiple bioactive gibberellins (GA₁, GA₃, GA₄) and abscisic acid (ABA) were significantly elevated in the Golden stage compared to the YG stage. Exogenous application of GA₃ and ABA in combination suppressed regreening and maintained the golden phenotype. This co-treatment reproduced the transcriptional signature of the Golden stage by upregulating the chlorophyll degradation gene *NYC-like* while downregulating both the carotenoid biosynthesis gene *PSY3* and cleavage genes *CCDs*.

**Conclusions:**

These findings demonstrate that the Golden stage in *U. pumila* ‘Jinye’ represents a state of developmental arrest synergistically maintained by elevated GA and ABA levels. This hormonal environment establishes a transcriptional program that maintains pigment metabolism into a high carotenoid/chlorophyll ratio and delays chloroplast maturation. Regreening results from the release of this arrest, coupling pigment metabolic reprogramming with the resumption of chloroplast development. This conceptual framework advances our understanding of leaf color regulation in woody plants and identifies GA/ABA signaling as potential targets for extending the ornamental phase of golden-leaf cultivars.

**Supplementary Information:**

The online version contains supplementary material available at 10.1186/s12870-026-09202-5.

## Introduction

Ornamental plants with diverse leaf colors are highly popular and play an indispensable role in landscaping. *Ulmus pumila* ‘Jinye’, a stable genetic bud mutant of *U. pumila*, exhibits bright golden leaves under field conditions, giving it considerable ornamental value [1–3]. Unlike species such as *Ginkgo biloba*, which only develop golden leaves in autumn, the tender leaves of *U. pumila* ‘Jinye’ maintain their golden color throughout the entire growing season. However, under field conditions, maturing leaves typically undergo a developmental transition known as “regreening” during which the golden hue gradually reverts to green [4–6]. A similar transition has been documented in the spathes of *Zantedeschia* [7]. In *U. pumila* ‘Jinye’, the natural regreening process shortens the ornamental phase, thereby diminishing its horticultural value and limiting its use in landscaping [1, 8]. Therefore, elucidating the regulatory mechanism underlying regreening in *U. pumila* ‘Jinye’ is crucial for stabilizing its golden leaf color and increasing its horticultural value.

Most colored-leaf plants, including *U. pumila* ‘Jinye’, originate from genetic mutations. In such mutants, the golden leaf phenotype is typically associated with chlorophyll deficiency, as reported for *Forsythia koreana* ‘Suwon Gold’ and *Lagerstroemia indica GL1* [4, 9]. Studies have indicated that leaf color formation depends on complex physiological processes, involving chlorophyll/carotenoid metabolism and chloroplast development [10, 11]. Consequently, these mutants serve as valuable materials for investigating fundamental mechanisms in photosynthesis, pigment metabolism, and chloroplast biogenesis [12, 13]. Plant pigments are a key factor in determining leaf color, which is defined by the type, content, and ratio of different pigments [14, 15]. In *U. pumila* ‘Jinye’, previous studies have confirmed lower contents of chlorophyll a, chlorophyll b, and total carotenoids compared to the wild-type *U. pumila*, but a higher carotenoid-to-chlorophyll ratio, which contributes to its golden appearance. These pigment alterations are often accompanied by physiological and ultrastructural modifications that can affect overall plant growth and performance [1].

To date, most studies on plant regreening have focused on fruits and flowers, whereas reports on leaves remain limited. For example, during fruit regreening in Valencia orange (*Citrus sinensis*), gibberellic acid (GA) significantly influences chlorophyll and carotenoid metabolism. It concurrently regulates key biosynthetic genes, such as *CAO*, *PAO*, and *SGR* for chlorophyll, and *PSY*, *PDS*, and *ZDS* for carotenoids [16, 17]. Blue LED light treatment also promotes regreening in Valencia orange flavedo by modulating the expression of *LCYe* and *CYb2*, shifting the carotenoid pathway toward the β, ε-branch [18]. In *Zantedeschia* spathes (a non-leaf organ), regreening involves chloroplast redifferentiation and chlorophyll reaccumulation, a process enhanced by 6-benzylaminopurine and GA₃ [7, 19, 20]. Despite these insights, the molecular basis of developmentally regulated leaf regreening in woody plants, particularly the role of phytohormones in natural mutants like *U. pumila* ‘Jinye’, remains largely unexplored.

Natural yellow leaf mutants in species such as *L. indica* [21, 22], *Oryza sativa* [23], and *Quercus shumardii* [24] provide valuable materials for exploring gene function and regulatory mechanisms. Integrated transcriptomic and metabolomic analyses have proven effective in elucidating the genetic and metabolic networks underlying complex biological processes [25, 26]. However, this integrated multi-omics approach has not been applied to achieve a systematic understanding of the regulatory networks underlying regreening in *U. pumila* ‘Jinye’. In this study, transcriptomic and metabolomic profiles were compared between two leaf color stages to capture the key molecular shifts using a multi-omics approach. This analysis was complemented by physiological assessments and a parallel investigation into phytohormonal regulation, which encompassed both endogenous quantification and exogenous application assays. Collectively, the integrated findings reveal the regulatory network underlying leaf regreening and provides a theoretical foundation for stabilizing ornamental traits in golden-leaf trees.

## Results

### Leaf color transition and pigment accumulation during regreening

**Fig. 1:**
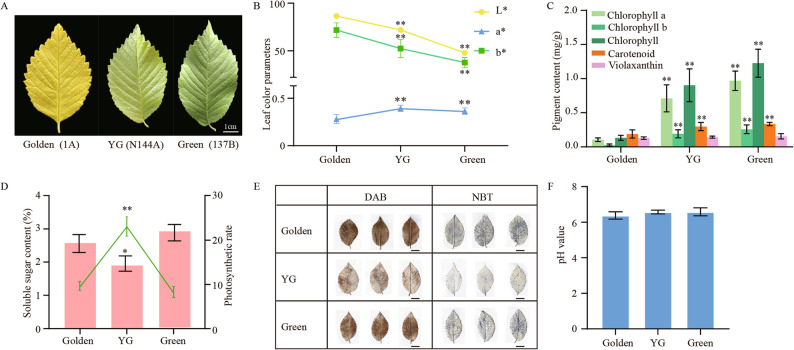
Physiological and histochemical characteristics of *U. pumila* ‘Jinye’ across the Golden, Yellowish-Green (YG), and Green stages. **A** Leaf color characteristics based on the Royal Horticultural Society (RHS) Large Colour Chart. **B** leaf color parameters. Data are shown as the mean ± SD of fifteen biological replicates. Commission Internationale de l’Éclairage: L* represents lightness, a* represents green–red axis, b* represents blue–yellow axis. **C** Photosynthetic pigment content. Data are shown as the mean ± SD of fifteen biological replicates. **D** Soluble sugar content and photosynthetic rate. Data are shown as the mean ± SD of three biological replicates. **E** Reactive oxygen species (ROS) staining. Staining was performed using NBT and DAB. Data are shown as the mean ± SD of three biological replicates. **F** Leaf pH value. Data are shown as the mean ± SD of three biological replicates. Statistical significance was determined by Student’s *t*-test, *, *p* < 0.05, **, *p* < 0.01

Under field conditions, the leaves of *U. pumila* ‘Jinye’ undergo a distinct regreening process during growth and development. Based on the Royal Horticultural Society (RHS) Large Colour Chart (Fig. 1A, Table S1) under standardized daylight conditions (10:00 AM–11:00 AM Beijing time), this process can be categorized into three stages: (i) Golden stage, (ii) Yellowish-Green (YG) stage, and (iii) mature Green stage (Fig. 1A). Color parameters were measured on the third fully expanded leaf from the shoot apex using an automatic colorimeter. Measurements were conducted in the CIE L* a* b* color space (Commission Internationale de l’Éclairage; L*: lightness, a*: green–red axis, b*: blue–yellow axis).

Comparative analysis showed significant differences (*p* < 0.01) in L*, a*, and b* values among the three stages. The Golden stage exhibited significantly higher L* and b* values (*p* < 0.01), consistent with its bright yellow appearance (Fig. 1B). These findings illustrated a clear progression in leaf color from Golden through the YG transitional stage to Green. To quantify pigment composition across these stages, the contents of total chlorophyll, chlorophyll a, chlorophyll b, carotenoids, and anthocyanin were measured. Pigment analysis indicated significant increases (*p* < 0.01) in all components except anthocyanin, with the highest levels observed in the Green stage (Fig. 1C). Moreover, the ratios of chlorophyll a to carotenoid, chlorophyll b to carotenoid, and total chlorophyll to carotenoid all gradually increased (*p* < 0.01) from the Golden to the Green stage, whereas the chlorophyll a/b ratio decreased (Table 1). These results indicated that the regreening process in *U. pumila* ‘Jinye’ was characterized by strong chlorophyll accumulation against the carotenoid background.

**Table 1:** Photosynthetic pigment ratios in *U. pumila* ‘Jinye’ across leaf color development stages

Ratio	Leaf color stages		
	Golden	YG	Green
Chlorophyll a/ Chlorophyll b	6.94 ± 1.18	4.08 ± 0.96*	3.04 ± 0.45**
Chlorophyll a/ Carotrnoid	0.58 ± 0.11	2.38 ± 0.27**	2.88 ± 0.22**
Chlorophyll b/ Carotrnoid	0.15 ± 0.08	0.67 ± 0.25**	0.76 ± 0.14**
Total chlorophyll/ Carotrnoid	0.72 ± 0.16	3.05 ± 0.46**	3.64 ± 0.36**

Data are expressed as the mean ± SD (*n* = 15). Statistical significance was determined by Student’s *t*-test

*YG* Yellowish-Green

* *p* < 0.05

** *p* < 0.01

### Physiological and biochemical characteristics during the transitional stage

The leaf color development of yellow-leaf mutants is usually related to changes in physiological and biochemical pathways [27, 28]. To investigate these changes in *U. pumila* ‘Jinye’, including photosynthetic rate, soluble sugar content, reactive oxygen species (ROS) accumulation (using DAB and NBT staining), and cellular pH were quantified across the regreening phase.

The YG transitional stage showed the highest photosynthetic activity (*p* < 0.01). Concurrently, soluble sugar content decreased significantly (*p* < 0.05) from the Golden to the YG stage (Fig. 1D). Histochemical staining revealed distinct levels of ROS accumulation across three stages (Fig. 1E). The strongest staining signals, indicating high ROS levels, were observed in the Golden stage. In contrast, the YG stage showed minimal staining, suggesting a strong scavenging capacity, while the Green stage exhibited an intermediate level. In addition, leaf pH remained stable across all stages, ranging from 6.5 to 7.3, with no significant differences observed (Fig. 1F).

These results indicate that the YG stage is an important transition period during regreening, characterized by active reorganization of physiological and biochemical processes defined by peak photosynthetic rate, reduced soluble sugar levels, and enhanced ROS scavenging capacity.

### Coordinated histological and ultrastructural modifications

**Fig. 2:**
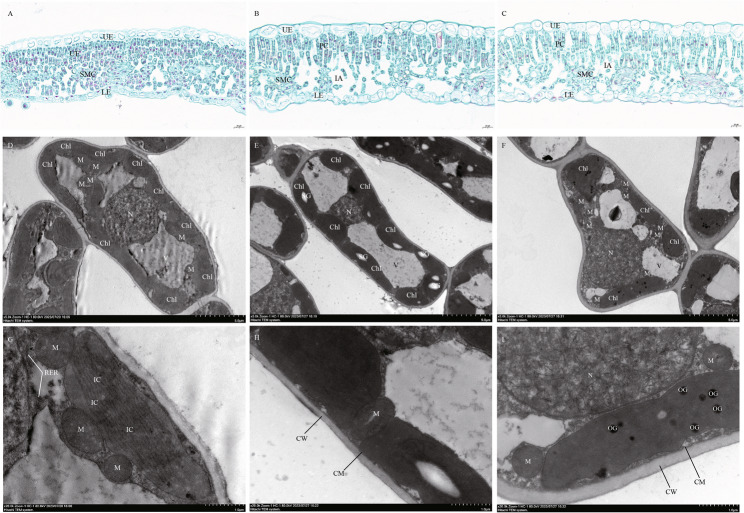
Anatomical and ultrastructural analysis of *U. pumila* ‘Jinye’ across leaf color development stages. **A**-**C** Paraffin sections showing leaf anatomy at the Golden, YG, and Green stages, respectively. UE, upper epidermis; LE, lower epidermis; PC, palisade cell; SMC, spongy mesophyll cell; IA, intercellular air space. **D**-**F** Transmission electron micrographs showing overall cellular ultrastructure at the Golden, YG, and Green stages, respectively. Scale bar = 5.0 μm. (G-I) Higher magnification views of chloroplasts and mitochondria from the corresponding stages, revealing detailed organellar morphology. Scale bar = 1.0 μm. CM: cell membrane; CW: cell wall; Chl: chloroplast; IC: granum lamella; M: mitochondria; N: nucleus; OG: osmiophilic globule; RER: endoplasmic reticulum; V: vacuole; SG: starch granule

The phenotypic expression of leaf coloration is modulated by multilayered histological characteristics, including (i) epidermal cell morphology, (ii) palisade tissue organization, and (iii) plastid distribution and ultrastructure [22, 29]. Histological analysis of paraffin-embedded leaf sections from *U. pumila* ‘Jinye’ across three developmental stages revealed dynamic architectural changes during regreening (Fig. 2A-C). Quantitative measurements indicated that leaf thickness in the Green and YG stages was 16.81% and 16.76% greater, respectively, compared to the Golden stage (*p* < 0.01). Significant thickening was also observed in the upper epidermis, lower epidermis, and palisade tissue (all *p* < 0.01; Table 2). Transverse sections further showed progressive changes in mesophyll structure: the Golden stage exhibited tightly packed palisade and spongy parenchyma cells, whereas the YG and Green stages displayed gradually expanded intercellular spaces and reduced cell-to-cell connectivity.

**Table 2:** Anatomical thickness of leaf tissues in *U. pumila* ‘Jinye’ across leaf color development stages

Thickness (µm)	Leaf color periods		
	Golden	YG	Green
Upper Epidermis	22.75 ± 1.57	27.51 ± 2.65**	27.71 ± 3.39**
Lower Epidermis	15.05 ± 1.47	19.06 ± 2.49**	20.79 ± 3.80**
Leaf Thickness	136.18 ± 7.33	158.91 ± 2.40**	159.07 ± 3.14**
Palisade Cells	36.2 ± 2.85	63.55 ± 6.28**	67.53 ± 6.47**
Spongy Cells	56.02 ± 5.70	54.00 ± 2.24	47.52 ± 3.98

Data are expressed as the mean ± SD (*n* = 6). Statistical significance was determined by Student’s t-test

* *p* < 0.05

** *p* < 0.01

Transmission electron microscopy (TEM) revealed significant ultrastructural alterations in leaf mesophyll cells throughout the regreening process, with pronounced differences in chloroplast and mitochondrial architecture (Fig. 2D-F). At the Golden stage, chloroplasts were abundant and contained a uniform stroma, but their grana thylakoids were loosely stacked and poorly defined. Mitochondria displayed intact membranes and electron-dense matrices, but their cristae were disorganized. Overall cellular integrity was generally maintained, with intact plasma membranes, uniformly thick cell walls, and no signs of plasmolysis.

In the YG and Green stages, more pronounced ultrastructural alterations were observed. In the YG and Green stages, chloroplasts contained abundant, prominent starch grains indicative of active photosynthetic carbon assimilation, while their thylakoid membranes appeared partially less electron-dense and less sharply defined, suggesting that the chloroplasts are undergoing normal structural adjustments rather than losing their photosynthetic function. The chloroplast envelope remained intact, and the stroma was well preserved. Furthermore, the loss of definition in chloroplast thylakoid membranes also indicated a remodeling of the membrane system. Taken together, these findings demonstrate that the regreening transition is associated with a coordinated ultrastructural remodeling of chloroplasts and mitochondria, including partial thylakoid de-stacking and altered cristae organization, but does not eliminate photosynthetic function, as evidenced by persistent starch accumulation. 

### Transcriptional regulation promotes chlorophyll biosynthesis and represses carotenoid accumulation

To elucidate the underlying molecular mechanism of regreening in *U. pumila* ‘Jinye’ leaves, a comparative transcriptome analysis of the YG versus Golden stages was performed via RNA-seq (Table S2). Principal component analysis revealed significant transcriptomic divergence between these stages (PC1: 87.96% and PC2: 3.9%) (Fig. S1A), demonstrating stage-specific transcriptional regulation. A total of 38,919 unigenes were assembled, with 28,786 (73.96%) successfully annotated against public databases (Table S3). Differential expression analysis identified 13,125 differentially expressed genes (DEGs) in the YG versus Golden stage comparison (|log2Fold Change| ≥ 1, FDR < 0.01), comprising 6,452 upregulated and 6,673 downregulated genes (Fig. S1B, C). Subsequent KEGG pathway analysis revealed 125 significantly enriched pathways (FDR < 0.05), covering metabolism, genetic information processing, and environmental information processing (Fig. S1D).

Transcriptome analysis between the YG and Golden stages identified the top 20 significantly enriched metabolic pathways, forming a coordinated regulatory network underlying leaf regreening during development (Fig. S1E). Among these, significantly differentially expressed pathways included plant hormone signal transduction (ko04075) and brassinosteroid biosynthesis (ko00905). These pathways may play pivotal roles in initiating chloroplast development and rejuvenation by regulating key genes involved in chlorophyll and carotenoid biosynthesis. This activation, in turn, could actively drive the regreening process [30, 31]. Concurrently, terpenoid backbone biosynthesis (ko00900) was upregulated to supply essential precursors—isopentenyl pyrophosphate (IPP) and dimethylallyl pyrophosphate (DMAPP)—for carotenoid biosynthesis, thereby providing substrates necessary for carotenoid accumulation. Beyond hormonal regulation, the circadian rhythm pathway (ko04712) coordinated the diurnal expression patterns of photosynthesis-associated genes, synchronizing their transcription with light-dark cycles to optimize photosynthetic efficiency. Meanwhile, glutathione metabolism (ko00480) contributed to pigment stability by scavenging reactive oxygen species (ROS) to mitigate photo-oxidative stress, thus protecting newly synthesized chlorophyll and carotenoids from degradation.

**Fig. 3:**
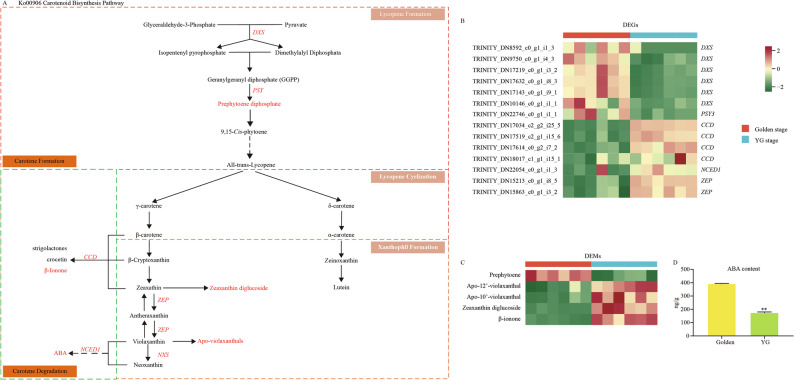
Integrated transcriptomic and metabolomic analysis of the carotenoid biosynthesis pathway in *U. pumila* ‘Jinye’ leaves between the Golden and YG stages. **A** Expression heatmap of unigenes mapped to the carotenoid biosynthesis pathway (ko00906). DEGs and DAMs are highlighted in red. **B** DEGs in the carotenoid biosynthesis pathway. Data are presented as FPKM values (fragments per kilobase of transcript per million mapped reads). Data are shown as the mean ± SD of six biological replicates. **C** DAMs in the carotenoid biosynthesis pathway. Data are shown as the mean ± SD of six biological replicates. **D** Abscisic acid (ABA) content in Golden and YG leaves measured by UPLC-ESI-MS/MS. Data are shown as the mean ± SD of three biological replicates. Statistical significance was determined by Student’s *t*-test, *, *p* < 0.05, **, *p* < 0.01

Within the carotenoid biosynthesis pathway (ko00906), transcriptomic analysis revealed significant differential expression of genes involved in both degradation and biosynthesis (Fig. 3A-B). Genes encoding *carotenoid cleavage dioxygenase* (*CCD*) enzymes were significantly upregulated during the YG stage, suggesting enhanced transcriptional activity driving CCD-mediated carotenoid cleavage. The concomitant induction of the *zeaxanthin epoxidase* (*ZEP*) gene, which encodes an enzyme for generating abscisic acid (ABA) precursors [32], and *9-cis-epoxycarotenoid dioxygenase 1* (*NCED1*), encoding the key rate-limiting enzyme in this pathway, further supports a metabolic shift toward ABA production. In contrast, a coordinated downregulation was observed in genes governing the upstream isoprenoid precursor supply and core carotenoid biosynthesis during the YG stage. Key genes in the MEP pathway, including the rate-limiting enzyme gene *1-deoxy-D-xylulose-5-phosphate synthase* (*DXS*), were significantly suppressed. Concurrently, *phytoene synthase 3* (*PSY3*) was markedly downregulated. In the early Golden stage, these genes (e.g., *PSY3*, *DXS*) were upregulated, providing substrates for carotenoid accumulation. In the later YG stage, these genes became downregulated, reducing substrate supply. This dynamic shift results in reduced net carotenoid accumulation at the YG stage, consistent with our metabolomic data. This synergistic transcriptional regulation, which involves the induction of oxidative cleavage and ABA biosynthetic genes and the repression of carotenoid biosynthetic genes, establishes a pattern consistent with reduced net carotenoid accumulation and an enhanced degradation flux.

**Fig. 4:**
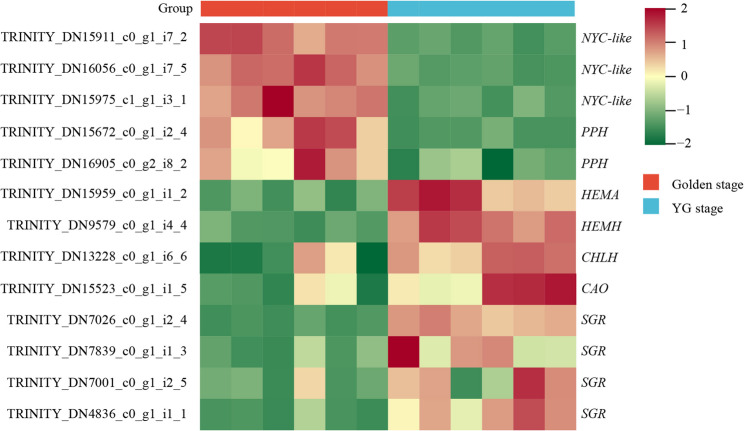
DEGs related to the chlorophyll metabolism pathway (ko00860). Data are presented as FPKM values. The data show six biological replicates

Analysis of DEGs in the porphyrin and chlorophyll metabolism pathway (ko00860) suggested its potential contribution to the regreening process (Fig. 4). Specifically, significant upregulation of the *HEMA* gene, which encodes the rate-limiting enzyme glutamyl-tRNA reductase (GluTR), may enhance metabolic flux into the tetrapyrrole pathway [24]. Concurrently, the marked downregulation of *non-yellow coloring*-*like* (*NYC*-*like*) genes in the YG stage, which encode key enzymes in chlorophyll catabolism, suggests that the initiation of chlorophyll degradation may be reduced [33]. Notably, although *SGR* shows a slight upregulation, the downstream catabolic genes *PPH*,* PAO*, and *RCCR* show no significant changes, and the *NYC*-*like* gene is strongly downregulated. As a result, overall chlorophyll degradation efficiency is not increased. Together with the upregulated chlorophyll biosynthesis genes, this contributes to net chlorophyll accumulation during regreening (Table S4). This coordinated regulation is likely to promote the retention and accumulation of chlorophylls, thereby potentially supporting chloroplast development and regreening. Furthermore, within the chlorophyll biosynthesis pathway itself, the upregulation of *CHLH* is predicted to enhance magnesium chelatase activity, while the upregulation of *HEMH* likely promotes heme biosynthesis [9, 34]. This coordinated regulation thus promotes chlorophyll synthesis and potentially reduces the accumulation of phototoxic intermediates like protoporphyrin IX. Additionally, the induction of *chlorophyllide a oxygenase* (*CAO*) is consistent with enhanced chlorophyll b biosynthesis, aligning with the observed decline in the chlorophyll a/b ratio. These transcriptional changes, including the coordinated upregulation of chlorophyll biosynthesis genes and the lack of activation of degradation pathway genes, are consistent with the net chlorophyll accumulation observed during regreening.

**Fig. 5:**
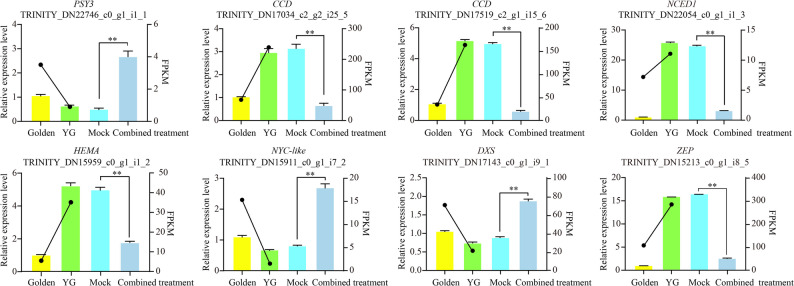
Expression of candidate genes in *U. pumila* ‘Jinye’ leaves across developmental stages and in response to combined GA₃+ABA treatment. Expression levels were normalized to *UpActin* and data are presented as the mean ± SD of three biological replicates. Statistical significance was determined by Student’s *t*-test, *, *p* < 0.05, **, *p* < 0.01

Collectively, the transcriptional profiles indicate that the YG stage is characterized by accelerated chlorophyll accumulation concurrent with reduced carotenoid accumulation, a molecular pattern consistent with our earlier pigment quantification results. Furthermore, qRT-PCR validation confirmed that the expression levels of key genes such as *PSY3*, *CCDs*, and *HEMA* were consistent with the RNA-seq data, corroborating the reliability of the sequencing results (Fig. 5). Together, these findings establish a clear causal logic chain: transcriptional activation of chlorophyll biosynthesis genes (e.g., *HEMA*, *CHLH*, *CAO*) coupled with repression of chlorophyll degradation genes (e.g., *NYC-like*) leads to net chlorophyll accumulation, while suppression of MEP pathway genes (e.g., *DXS*) and carotenoid biosynthetic genes (e.g., *PSY3*) combined with induction of cleavage-related genes (e.g., *CCDs*) leads to reduced carotenoid levels. This integrated transcriptional reprogramming thus provides the molecular basis for the observed metabolic shifts during regreening.

### Dynamic alterations in the metabolite profile support regreening

To systematically elucidate the metabolic basis of regreening suggested by the transcriptomic profiles, LC-MS-based non-targeted metabolomic profiling was conducted using leaf samples collected from the Golden and YG stages to characterize the dynamic alterations in the metabolomic profile during regreening (Fig. S2, Table S5). Principal component analysis (PCA) revealed a clear metabolic separation between the two stages, with the first principal component (PC1) accounting for 64% of the total variance (Fig. S2A). Comparative analysis identified 1507 differentially abundant metabolites (DAMs, VIP > 1 from PLS-DA model and *p* < 0.05), with 706 upregulated and 801 downregulated in the YG stage compared to the Golden stage (Fig. S2B).

Metabolomic analysis revealed the top 20 metabolic pathways with the most significant alterations in metabolite accumulation between the YG and Golden stages of *U. pumila* ‘Jinye’ (Fig. S2D). Several of these pathways were critically involved in pigment metabolism and leaf regreening. Specifically, pyruvate metabolism supplied essential carbon precursors for carotenoid biosynthesis, while alanine, aspartate, and glutamate metabolism provided glutamate—a key substrate for chlorophyll biosynthesis. The TCA cycle supplied energy and carbon skeletons to meet the biosynthetic demand, and ascorbate and aldarate metabolism mitigated photo-oxidative damage through ROS scavenging, together promoting pigment stability. Collectively, this metabolic reprogramming establishes a coordinated network that supports leaf regreening in *U. pumila* ‘Jinye’ by orchestrating precursor synthesis, energy supply, and antioxidant defense.

### Multi-omics analysis reveals transcriptional coordination underlying metabolic shifts in regreening

Building on prior findings that the regreening of *U. pumila* ‘Jinye’ leaves from the Golden to the YG stage is driven primarily by a shift in the chlorophyll-to-carotenoid ratio, an integrated analysis of DAMs and DEGs was performed to elucidate the regulatory mechanisms underlying this color transition, focusing on carotenoid and chlorophyll metabolism. A global correlation analysis between all identified DEGs and DAMs was conducted using Spearman’s rank correlation coefficient (Supplementary Table S6).

Within the carotenoid biosynthesis pathway (ko00906), 12 DEGs and 7 DAMs were systematically mapped to their corresponding enzymatic steps (Fig. 3A-C). A strong downregulation of the core carotenogenic gene *PSY3* was observed, concomitant with a pronounced decrease in its catalytic product, phytoene. Similarly, *DXS* genes, which encode rate-limiting enzymes in the MEP pathway, were significantly downregulated in the YG stage, reducing the precursor supply for carotenoid biosynthesis. Notably, the carotenoid degradation pathway was activated, evidenced by upregulation of *carotenoid cleavage dioxygenases* (*CCDs*). This led to elevated levels of their direct cleavage product, β-ionone, thus indicating accelerated carotenoid catabolism. Simultaneously, the expression of *NCED1* and *ZEP* were coordinately upregulated. ZEP catalyzes the synthesis of violaxanthin and neoxanthin [35, 36], which serve as substrates for NCED-mediated cleavage into ABA [37]. Consistent with this induction, downstream metabolites such as zeaxanthin diglucoside and apo-violaxanthals accumulated significantly during the YG stage. However, despite the coordinated upregulation of these upstream ABA biosynthetic genes, the severe deficiency of carotenoid precursors, caused by the downregulation of *PSY3* and *DXS*, ultimately led to a significant reduction in ABA accumulation (*p* < 0.01). Accordingly, LC-MS/MS analysis showed that endogenous hormone ABA levels in the YG stage were reduced by 44.68% compared to the Golden stage (Fig. 3D).

Consequently, metabolic flux during regreening was redirected from carotenoid biosynthesis toward degradation. This shift was due to substantial downregulation of biosynthetic genes coupled with activation of degradation pathways. Concurrently, chlorophyll biosynthesis genes (*HEMA*, *CHLH*, *CAO*) were coordinately upregulated while degradation genes (*NYC-like*) were suppressed, despite no significant concurrent changes in chlorophyll metabolite levels being detected (Fig. 4). Collectively, this transcriptional reprogramming toward chlorophyll synthesis and away from carotenoid accumulation result in the marked shift in pigment balance underlying the regreening phenotype.

### Functional validation of GA and ABA crosstalk in arresting the regreening process

**Fig. 6:**
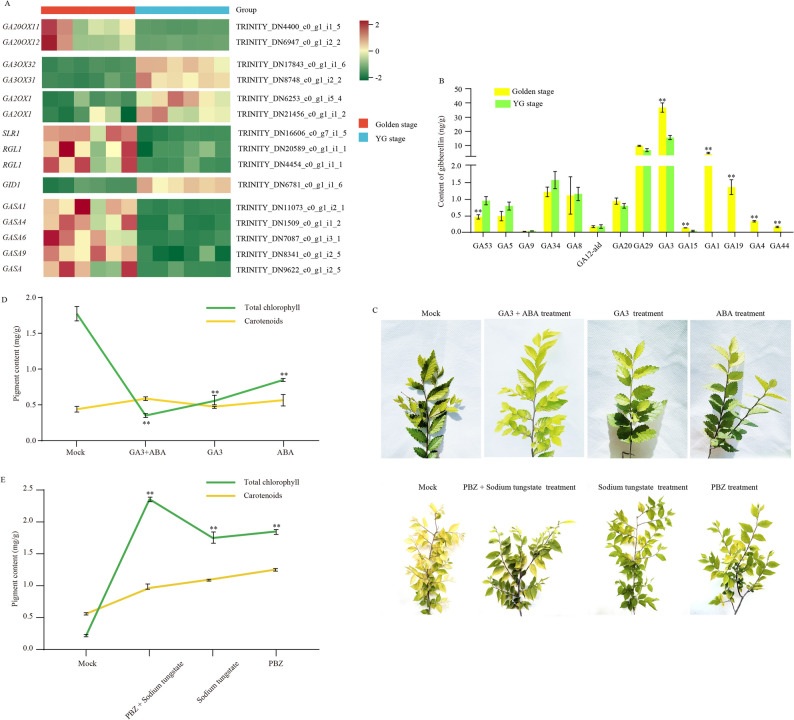
Effects of gibberellin and abscisic acid on leaf color regulation in *U. pumila* ‘Jinye’. **A** Transcriptional profiling of GA biosynthesis-related DEGs. Data are presented as FPKM values. Data are presented as the mean ± SD of six biological replicates. **B** Endogenous content of various bioactive GAs quantified by UPLC-ESI-MS/MS. Data are shown as the mean ± SD of three biological replicates. **C** Leaf color phenotypes in response to GA3 and ABA treatments. **D** Total chlorophyll and carotenoid content after phytohormone treatment. Data are shown as the mean ± SD of three biological replicates. Statistical significance was determined by Student’s t-test, *, *p* < 0.05, **, *p* < 0.01

Prior integrated transcriptomic and metabolomic analyses suggested a strong association between the regreening process of *U. pumila* ‘Jinye’ and phytohormone dynamics. Therefore, the analysis was focused on hormone-related DEGs. Investigation of these DEGs revealed that the gibberellin (GA) metabolic and signaling pathway was markedly changed (Fig. 6A). The results showed that key GA metabolism genes, including biosynthetic genes *GA20OX*, *GA3OX*, and the catabolic gene *GA2OX*, were significantly differentially expressed between the Golden and YG stages. Specifically, expression of GA signaling repressors *RGL1* and *SLR1* was upregulated in the Golden stage, whereas expression of *GID1*, which encodes the GA receptor, was downregulated. This expression pattern is consistent with a homeostatic feedback response to elevated endogenous GA levels, wherein increased repressor expression and decreased receptor expression suppress GA signaling. Consistent with the elevated GA levels, transcript levels of GASA genes, which encode typical GA-responsive proteins, were significantly higher in the Golden stage than in the subsequent YG stage (Fig. 6A). To validate these molecular changes at the hormonal level, endogenous gibberellin levels were quantified by LC–MS/MS. The analysis showed that the concentrations of multiple GAs, including GA₁, GA₃, GA₄, GA₁₅, GA₁₉, and GA₄₄, were significantly higher in the Golden stage than in the YG stage (Fig. 6B). In particular, GA₁, GA₄, GA₁₉, and GA₄₄ were detectable in the Golden-stage tissues but fell to undetectable or trace levels in the YG stage samples (detection limits: 2 ng/mL for GA₁ and GA₄; 5 ng/mL for GA₁₉ and GA₄₄).

As quantified above (Fig. 3D), endogenous levels of both GA and ABA decline from the Golden to the YG stage. The coordinated decline suggests that the high-GA and high-ABA hormonal environment is associated with the arrested golden leaf state. To test the functional role of this hormonal environment, we hypothesized that exogenous co-application of GA₃ and ABA during the early Golden stage would maintain this arrested state, thereby delaying regreening. Therefore, Golden stage leaves were treated with GA₃ (300 mg/L), ABA (20 mg/L), or a combination of both by foliar spray. Treatments began at the start of the early Golden stage and were repeated every three days for a two-week period to cover the critical color-transition phase. Phenotypic analysis showed that only the combined application of GA₃ and ABA significantly attenuated the regreening (Fig. 6C), suggesting a synergistic role of these hormones. Consistently, pigment measurements showed that chlorophyll content was most severely suppressed under the combined treatment, reaching only 20.03% of the mock-treated control (sprayed with solvent only). This reduction was significantly greater than that caused by individual GA₃ (31.54% of control) or ABA (49.71% of control) treatments (Fig. 6D). In contrast, carotenoid content showed no significant difference among all the treatments, ranging from 1.08 to 1.33-fold of the control. Consequently, the total chlorophyll/carotenoid ratio in the co-treated leaves was significantly reduced to 0.60, representing only about 15% of the control value (3.89) (Table S7). These results demonstrate that the synergistic co-application of GA₃ and ABA is required to strongly inhibit chlorophyll accumulation and thus arrest the regreening process. To further test whether endogenous GA and ABA are required to maintain the golden leaf phenotype, we sprayed the GA biosynthesis inhibitor paclobutrazol (PBZ, 300 mg/L) and the ABA biosynthesis inhibitor sodium tungstate (200 mg/L) onto leaves at the golden stage. Inhibition of either GA or ABA biosynthesis significantly accelerated the regreening process, and combined inhibition of both showed an even stronger effect, as evidenced by increased total chlorophyll content reaching 10.85‑fold of the mock control and an increased chlorophyll‑to‑carotenoid ratio reaching 2.55 (the mock control ratio was 0.39), phenocopying the natural transition from the golden to the YG stage (Fig. 6E, Table S8). These results provide complementary evidence that endogenous GA and ABA act synergistically to arrest regreening and maintain the golden leaf color. 

Subsequent qRT-PCR analysis revealed that the co-application of GA₃ and ABA significantly altered the expression of key genes in the pigment metabolic pathways on the 7th day of treatment (Fig. 5). The chlorophyll catabolism gene *NYC-like* was significantly upregulated, whereas the expression of *HEMA* remained unchanged compared to the mock control. The sustained upregulation of *NYC-like* under co-treatment likely maintained a high rate of chlorophyll degradation, thereby contributing to the preservation of the golden leaf phenotype and attenuation of regreening. Furthermore, genes associated with carotenoid degradation, such as *CCDs*, were significantly downregulated. Notably, the expression of *PSY3*, which encodes the rate-limiting enzyme for carotenoid biosynthesis, was also significantly downregulated. This coordinated transcriptional downregulation of both biosynthesis (*PSY3*) and degradation (*CCDs*) genes would stabilize existing carotenoid levels, thereby maintaining yellow pigmentation.

Collectively, these results provide functional evidence that the synergistic action of GA₃ and ABA establishes a specific transcriptional program responsible for arresting leaf development, and effectively maintaining the golden leaf phenotype. This mechanism is consistent with the elevated endogenous levels in the Golden stage and directly explains the coordinated metabolic changes described above.

## Discussion

Leaf color mutants are valuable models for studying pigment metabolism, chloroplast development, and plant environmental adaptation [12, 13, 38]. This study investigated the developmental regreening process in *U. pumila* ‘Jinye’, a golden-leaf mutant that transitions from bright golden to green during leaf maturation. By integrating physiological, transcriptomic, metabolomic, and hormonal analyses, a coordinated regulatory network underlying this color transition was revealed. Our findings provide an integrated framework for understanding regreening, demonstrating that it is not merely a consequence of pigment accumulation but a programmed developmental process involving structural reorganization, metabolic reprogramming, and phytohormone interactions.

The regreening phenotype in *U. pumila* ‘Jinye’ is accompanied by a clear change in pigment balance. Physiological data showed that the transition from the Golden to the Green stage involves a significant increase in chlorophyll content and a relative decrease in carotenoids, resulting in a rising chlorophyll-to-carotenoid ratio (Sect. 2.1). This finding aligns with the classic observation in golden-leaf mutants such as the yellow-leaf variety of *Quercus shumardii* (the Shumard oak) [24]. Despite these valuable insights, several limitations should be acknowledged. We have not yet established a quantitative threshold for the carotenoid/chlorophyll (Car/Chl) ratio in relation to the leaf color transition. Although data from three developmental stages (Golden, YG, and Green) are available, the limited number of sampling time points precludes the derivation of a reliable threshold or a robust statistical correlation. Therefore, future studies should conduct continuous, year‑round sampling throughout the growing season to more comprehensively analyze the relationship between the Car/Chl ratio and the regreening process, with the ultimate goal of establishing a potential threshold. Addressing this question would further strengthen the predictive power of pigment dynamics as an indicator of developmental stage and enhance the practical applicability of our findings for breeding and horticultural management. Furthermore, transcriptomic analyses indicate that the process is actively regulated at the gene expression level. As this study demonstrates, a coordinated transcriptional regulation (Sect. 2.4) suppresses carotenoid biosynthesis while promoting chlorophyll production, underscoring the transcriptional control of pigment metabolism during regreening. Specifically, the gene expression profile of the key YG transitional stage, compared to the Golden stage, shows upregulation of key chlorophyll biosynthesis genes (e.g., *HEMA*, *CHLH*, *CAO*) and downregulation of chlorophyll degradation genes (e.g., *NYC-like*), thereby facilitating net chlorophyll accumulation. Notably, despite these clear transcriptional changes, no significant accumulation of chlorophyll pathway metabolites was detected in the metabolomic data. This mismatch likely reflects a combination of factors. It may reveal a delay between transcriptional activation and the subsequent processes of protein translation, post-translational modification, and assembly into functional enzyme complexes, meaning the transcriptional upregulation has not yet fully manifested as immediate metabolite accumulation. This delay is further compounded by a highly efficient biosynthetic system characterized by rapid turnover of low-abundance metabolic intermediates, along with potential post-translational regulation and the inherent technical challenges of detecting transient metabolites. Therefore, rather than indicating low metabolic activity, these findings suggest a tightly regulated metabolic flux that efficiently directs resources toward chlorophyll production without significant intermediate buildup, thereby supporting the high chlorophyll demand during regreening while maintaining overall metabolic homeostasis.

Concurrently, the carotenoid metabolic pathway is redirected from accumulation toward degradation. This shift is evidenced by the significant downregulation of core biosynthetic genes (*PSY3*, *DXS*) coupled with the upregulation of cleavage enzymes (*CCDs*) [39]. Within this redirected carotenoid metabolism, the induction of *ZEP* and *NCED1* suggests a concurrent push toward ABA biosynthesis [32, 40]. However, the substantial reduction in carotenoid precursor pools ultimately limits ABA production [37, 41], leading to its decreased abundance in the YG stage. Notably, the upregulation of terpenoid backbone biosynthesis and pyruvate metabolism does not necessarily contradict the reduced carotenoid accumulation, because the terpenoid backbone pathway supplies precursors not only for carotenoids but also for other terpenoids, including the phytol tail of chlorophyll, sterols, and various phytohormones. Thus, the increased flux may support chlorophyll synthesis and other metabolic demands during regreening rather than carotenoid accumulation. Together, this intricate transcriptional control ensures a metabolic flux that favors chlorophyll synthesis over carotenoid accumulation, directly contributing to the observed color change.

This metabolic change was accompanied by significant anatomical and ultrastructural modifications. The thickening of the leaf blade, especially the palisade tissue, during the YG and Green stages suggests a developmental progression toward improved light interception and mesophyll gas exchange, which may contribute to the elevated whole‑leaf photosynthetic rate measured at the YG stage despite concurrent ultrastructural remodeling [9, 42]. Observations by transmission electron microscopy further revealed substantial subcellular changes. The loosely stacked thylakoids in the Golden stage chloroplasts suggest an immature or impaired photosynthetic apparatus, consistent with the bright yellow coloration and presumably lower photosynthetic capacity, as also reported for the yellow-green rice mutant *ygl16* [24]. Notably, the YG stage, despite showing a net accumulation of chlorophyll, exhibited the lowest photosynthetic rate. This may be attributed to the observed blurring of thylakoid membranes during this transitional period, indicating that the functional maturation of the photosynthetic apparatus lags behind pigment biosynthesis and is concomitant with a decline in membrane integrity. Progressing to the Green stage, chloroplasts accumulated prominent starch grains, signifying active carbon fixation; however, the persistence of blurred thylakoid membranes and degenerated mitochondrial cristae indicated that structural refinement of organelles was incomplete. Our ultrastructural observations revealed that the leaf color transition is accompanied by a gradual decline in the integrity of organelle structures, particularly the thylakoid membranes and mitochondrial cristae. These progressive alterations are closely associated with the developmental process of leaf color transition. The coexistence of early senescent features, such as blurred thylakoid membranes and regressed mitochondria [43], implies that senescence initiates shortly after regenerative maturation, with supporting evidence from other yellow-leaf mutants [21, 44]. These ultrastructural changes demonstrate that regreening is a transient, coordinated cellular state in which the processes of organellar maturation and early senescence coexist to support the metabolic demands of leaf development. The high net photosynthetic rate measured in YG leaves seems to conflict with the observation that their thylakoid membranes appear partially loose and less well‑defined. This apparent contradiction can be resolved by considering several factors. The blurred appearance of thylakoid membranes does not indicate a loss of photosynthetic function, as evidenced by the abundant starch grains in these chloroplasts that clearly show active carbon fixation. Moreover, YG leaves possess significantly thicker palisade tissue and enlarged intercellular spaces (Table 2), which enhance light interception and CO₂ diffusion, thereby boosting whole‑leaf photosynthesis even while individual chloroplasts undergo structural remodeling. In addition, the disorganized mitochondrial cristae likely reflect normal metabolic adjustments during leaf development, such as increased photorespiration or nitrogen reassimilation, rather than direct photosynthetic impairment. Such partial dissociation between local organelle structure and overall leaf function is actually common during the transition from a young, nutrient‑importing leaf to a mature, photosynthetically active leaf. Therefore, the high photosynthetic rate in YG leaves is fully compatible with the observed ultrastructural changes [45].

Beyond changes in pigment metabolism, our findings underscore the importance of reactive oxygen species (ROS) homeostasis during regreening. Histochemical staining revealed substantial ROS accumulation in the Golden stage, indicating photo-oxidative stress associated with chlorophyll deficiency. This high level of ROS was effectively alleviated in the YG stage, implying the activation of efficient antioxidant systems [46]. Intriguingly, the strongest ROS signal was observed in the mature Green stage, which may signal the initiation of leaf senescence [47]. Consistent with the need for ROS scavenging in the YG stage, transcriptome data showed enrichment of components involved in glutathione metabolism, a major pathway for ROS scavenging. The enhanced antioxidant capacity likely protects newly synthesized chlorophyll and supports chloroplast development by mitigating oxidative damage. In parallel, the decrease in soluble sugar content from the Golden to the YG stage may be attributed to their increased utilization as energy sources and carbon skeletons for chloroplast development and pigment synthesis [48, 49]. The combination of low photosynthetic rate and high biosynthetic demand during the YG stage provides a coherent explanation for the observed decline in soluble sugar content.

One of the most significant findings of this study is the elucidation of a synergistic hormonal module involving gibberellin and ABA in arresting the regreening process. Integrated analysis identified phytohormone signal transduction as one of the most significantly altered pathways. The Golden stage is characterized by elevated endogenous levels of multiple bioactive GAs (e.g., GA₁, GA_3_, GA_4_) and ABA. This hormonal environment is associated with a gene expression pattern that suggests an active feedback control mechanism for GA signaling [50]. Specifically, the upregulation of GA-signaling repressor genes, *RGL1* and *SLR1*, coupled with the downregulation of the GA receptor gene *GID1* under high endogenous GA conditions represents a compensatory response to reduce GA signaling activity and maintain homeostasis [51, 52]. To further elucidate the connection between these hormonal signals and pigment metabolism genes, the expression of key transcription factors integrating GA and ABA signaling was examined. Transcriptome data revealed that one *phytochrome-interacting factor* (*PIF*) gene, which act as central hubs integrating hormonal and environmental signals, were significantly upregulated in the Golden stage compared to the YG stage (Table S9). Concurrently, abscisic acid insensitive 5 (*ABI5*), a core transcription factor in ABA signaling that can be activated by PIFs, also showed elevated expression. This expression pattern suggests a potential regulatory cascade in which elevated GA and ABA in the Golden stage promote *PIF* accumulation, which in turn may activate *ABI5* expression. Studies in Arabidopsis have shown that PIFs directly bind G-box elements in the promoters of genes encoding chlorophyll biosynthesis enzymes and repress their expression. Additionally, *PIFs* have been demonstrated to directly suppress *PSY* expression, the rate-limiting enzyme in carotenoid biosynthesis [53]. Therefore, the high *PIF* and *ABI5* expression in the Golden stage may directly contribute to the observed transcriptional program—suppressing chlorophyll synthesis while stabilizing carotenoid levels—thereby maintaining the golden phenotype. Functional validation through exogenous hormone application confirmed that only the combined treatment of GA₃ and ABA significantly attenuated regreening and maintained the golden phenotype, demonstrating a synergistic effect mediated by the specific transcriptional regulation of pigment metabolism. The GA₃ and ABA co-treatment upregulated the chlorophyll catabolism genes *NYC-likes* while suppressing for carotenoid degradation genes (e.g., *CCDs*) and biosynthesis gene *PSY3*. *PSY3* catalyzes the first committed step of carotenoid biosynthesis, the condensation of GGPP into phytoene [54]. This coordinated gene expression pattern would promote chlorophyll breakdown while stabilizing the existing carotenoid pool, thereby resulting in a markedly reduced chlorophyll-to-carotenoid ratio and the preservation of the golden leaf color. These results suggest that the high endogenous levels of GA and ABA in the Golden stage create a hormonal environment that inhibits the developmental progression toward regreening, possibly by restricting full chloroplast maturation. This discovery is consistent with the role of GA and ABA in regulating pigment dynamics in other systems, such as fruit regreening in citrus [31], and extends this paradigm to a developmentally regulated leaf color transition in a woody perennial. Whether this synergistic interaction represents a general mechanism in woody plant leaf development or a specific feature of the ‘Jinye’ mutant remains to be determined. This mode of GA and ABA synergy in regulating leaf color transition has not been reported in other woody plants, suggesting it may be unique to the ‘Jinye’ genetic background. Furthermore, the observed synergy between GA and ABA in arresting regreening presents a compelling case of context-dependent hormone interaction, which contrasts with their canonical antagonism in processes such as seed germination.

Specifically, leaf color transition may be influenced by factors such as light intensity (shading stress) and temperature (heat stress), as previously reported in this species [1, 5, 8]. However, in the present study, all samples were collected during the same growing season (April to September), representing three distinct developmental stages (Golden, YG, and Green), and were taken from the same field plot under identical environmental conditions. We deliberately chose this experimental design to minimize the interference of environmental heterogeneity, thereby allowing us to focus on the endogenous developmental regulation of leaf color transition. Therefore, we believe that the observed leaf color changes are primarily driven by developmental programs rather than by external environmental cues.

Beyond providing mechanistic insights into hormone regulation, this study reveals practical applications for manipulating leaf color through silvicultural practices. Combined exogenous hormone application effectively prevents regreening in *U. pumila* ‘Jinye’, making hormone spraying a promising short-term silvicultural approach for managing the leaf color of golden-leaf plants. Relative to traditional environmental regulation strategies, such as modifying water and fertilizer regimes, adjusting light conditions, or pruning, hormone treatment is more feasible, less costly, and yields stronger effects. Moreover, this technique supports rapid, directed changes in leaf coloration and enables fine-tuned control via adjustments in concentration and application timing.

## Conclusion

This study provides the first evidence in a woody perennial that gibberellin and abscisic acid synergistically regulate leaf color transition through a developmental arrest mechanism. In *Ulmus pumila* ‘Jinye’, the Golden stage represents a state of hormone-mediated arrest, characterized by elevated endogenous GA and ABA levels, delayed chloroplast maturation, and a transcriptional program maintaining a high carotenoid/chlorophyll ratio. The decline of these hormones during development releases this arrest, initiating coordinated metabolic changes in which upregulation of chlorophyll biosynthesis genes (*HEMA*, *CHLH*, *CAO*) coupled with downregulation of degradation genes (*NYC-like*) leads to net chlorophyll accumulation, while suppression of carotenoid biosynthesis genes (*DXS*, *PSY3*) combined with induction of cleavage genes (*CCDs*) limits carotenoid accumulation. This coordinated transcriptional shift directly explains the elevated chlorophyll/carotenoid ratio underlying the regreening phenotype. Ultrastructural evidence reveals that this transition involves both chloroplast maturation and early senescence features, indicating regreening is a dynamic cellular state where developmental progression and structural degeneration coexist. Exogenous hormone validation confirms that GA and ABA act synergistically to maintain the arrested state through direct transcriptional regulation of pigment metabolism genes. These findings establish a conceptual framework for understanding leaf color regulation in woody plants, demonstrating that golden coloration can result from active hormonal maintenance of developmental arrest rather than passive pigment deficiency. The identified GA/ABA synergistic module offers potential targets for extending the ornamental phase of golden-leaf cultivars. Future studies should investigate the upstream signals regulating these hormonal changes and explore whether similar mechanisms operate in other woody plant leaf color mutants.

## Materials and methods

### Plant materials

Leaf samples of *U. pumila* ‘Jinye’ were collected from the nursery base of Hebei Normal University of Science & Technology (39°N, 119°E). Samples representing different developmental stages were collected monthly from April to September during the growing season. All the leaf samples were immediately frozen in liquid nitrogen and stored at − 80 °C until used for transcriptomic, metabolomic, and physiological analyses.

### Quantification of leaf coloration and photosynthetic pigment content

Leaf color was quantified using both the Royal Horticultural Society Colour Chart (RHSCC) and a colorimeter (CR-400, Konica Minolta, Japan). Measurements were taken with 15 biological replicates, and the CIE L, a*, and b* values were recorded [55]. The contents of chlorophyll, carotenoid, and anthocyanin were determined according to the method of Li (2000), with three biological replicates for each assay. The following formulas were applied:

$$\text{Chlorophyll a}\;\left(\mathrm{mg/g}\right)=0.1\times\left(9.78\times\mathrm{OD}_{663}-0.99\times\mathrm{OD}_{645}\right)$$

$$\text{Chlorophyll b}\;\left(\mathrm{mg/g}\right)=0.1\times\left(21.43\times\mathrm{OD}_{645}-4.78\times\mathrm{OD}_{663}\right)$$

$$\text{Total chlorophyll}\;\left(\mathrm{mg/g}\right)=0.1\times\left(5.13\times\mathrm{OD}_{663}-20.44\times\mathrm{OD}_{645}\right)$$

$$\mathrm{Carotenoid}\;\left(\mathrm{mg/g}\right)=0.1\times\left(4.7\times\mathrm{OD}_{440}-0.27\times\text{total chlorophyll}\right)$$

$$\mathrm{Anthocyanin}\;\left(\mathrm{mg/g}\right)=\mathrm{OD}_{535}/\left(0.1\times\text{fresh weight}\right)$$

### Measurement of net photosynthetic rate, pH, and soluble sugar content

The net photosynthetic rate was measured using a portable photosynthesis system (LI-6400XT, LI-COR, Lincoln, NE, USA) on clear days between 9:00 AM and 11:00 AM. For each of the three biological replicates, a fully expanded, sun-exposed leaf was clamped in the standard leaf chamber (2 cm × 3 cm) for measurement. The pH value of leaf homogenates was measured directly using a pH meter. The soluble sugar content was quantified using the anthrone-sulfuric acid colorimetric method [56]. Each assay was performed with three independent biological replicates.

### Anatomical and ultrastructural observation

Light microscopy observation: Leaf samples of *U. pumila* ‘Jinye’ were fixed in FAA solution, followed by dehydration, clearing, wax impregnation, and paraffin embedding. Sections were prepared using a microtome (RM2235, Leica, Germany), double-stained with Safranin O and Fast Green, and mounted with neutral resin prior to observation under a light microscope (Eclipse E100, Nikon, Japan). The thickness of the palisade tissue, spongy tissue, entire leaf blade, upper epidermis, and lower epidermis was measured using accompanying imaging software (NIS-Elements D, Nikon, Japan).

Transmission electron microscopy observation: Fresh leaf samples were immediately fixed in 4% glutaraldehyde solution. Vacuum infiltration was applied to ensure complete penetration of the fixative. When fixing the sample, apply a vacuum pressure of 0.05–0.1 MPa was applied for 30 min until the sample completely sink to the bottom. After fixation, samples were rinsed, dehydrated, and embedded in epoxy resin. Ultrathin sections were prepared using an ultramicrotome (EM UC7, Leica, Germany), stained with uranyl acetate and lead citrate, and examined under a transmission electron microscope (HT7800, Hitachi, Japan) to observe the intracellular ultrastructure.

### Transcriptome Sequencing and Bioinformatic Analysis

Total RNA was extracted from *U. pumila* ‘Jinye’ leaf samples using the RNAprep Pure Plant Plus Kit (TIANGEN, Beijing, China), with six biological replicates. RNA integrity, concentration, and purity were assessed using agarose gel electrophoresis and a spectrophotometer (Nanodrop). Sequencing libraries were constructed and sequenced on an Illumina HiSeq™ 2500 platform (Illumina, San Diego, CA, USA). Raw reads were processed to remove low-quality sequences and adapters using Trimmomatic to obtain clean reads. De novo transcriptome assembly was then performed using Trinity software with default parameters. All assembled transcripts were aligned against the NCBI non-redundant (Nr) protein database for functional annotation and unigene identification. Gene Ontology (GO) enrichment and Kyoto Encyclopedia of Genes and Genomes (KEGG) pathway analyses were conducted on differentially expressed genes (DEGs) to identify significantly enriched biological themes and metabolic pathways.

### Validation of RNA-Seq data by quantitative Real-Time PCR

To validate the RNA-seq results, DEGs were selected for quantitative real-time PCR (qRT-PCR) analysis, with three biological and three technical replicates. Total RNA was reverse transcribed into cDNA using the PrimeScript™ RT Reagent Kit with gDNA Eraser (Takara, Tokyo, Japan). The *U. pumila* Actin (UpActin) gene was used as an internal reference [9]. qRT-PCR was performed on a Bio-Rad CFX96 Real-Time PCR System using the UltraSYBR Mixture kit (CoWin, Jiangsu, China), following the manufacturer’s protocols. The relative expression levels of target genes were calculated using the 2⁻ΔΔCT method.

### Widely targeted metabolomic analysis

For metabolomic analysis, *U. pumila* ‘Jinye’ leaf samples were collected from six biological replicates. Metabolite profiling was conducted using an ultra-performance liquid chromatography-tandem mass spectrometry (UPLC-MS/MS) system (ACQUITY UPLC, Waters, USA; Q-Exactive, Thermo Fisher Scientific, USA). The reproducibility and inter-group variation were evaluated by PCA and sample correlation analysis based on the acquired mass spectrometry data. Differential metabolites between groups were identified by integrating orthogonal partial least squares-discriminant analysis (OPLS-DA) and univariate analysis (Student’s *t*-test). Metabolites exhibiting a variable importance in projection (VIP) > 1 from the OPLS-DA model and *p* < 0.05 from the *t*-test were considered significantly differentially expressed.

### Quantification of endogenous gibberellins and ABA by UPLC-ESI-MS/MS

We sprayed the GA₃ solution (300 mg/L) onto the leaves until runoff. The applied volume per plant varied slightly, but all treatments (control, GA₃, ABA, and GA₃+ABA, sodium tungstate, PBZ, sodium tungstate + PBZ) were sprayed using the same method and endpoint [57–59]. Leaf samples of *U. pumila* ‘Jinye’ were collected from three independent biological replicates. Endogenous gibberellins and ABA were extracted and purified, then analyzed using an ultra-performance liquid chromatography-electrospray ionization tandem mass spectrometry (UPLC-ESI-MS/MS) system (ExionLC™ AD, SCIEX, USA; QTRAP^®^ 6500+, SCIEX, USA). Quantification was performed by MetWare Biological Science and Technology Co., Ltd. (Wuhan, China) using multiple reaction monitoring (MRM) mode. Reference standards were used for identification and calibration of individual gibberellin compounds. 

## Supplementary Information

Supplementary Material 1.

Supplementary Material 2. Table S4. Differentially expressed genes in chlorophyll synthesis and metabolic pathways.

Supplementary Material 3.

Supplementary Material 4. Table S1. Analysis of leaf color development in U. pumila ‘Jinye’ based on the Royal Horticultural Society Large Colour Chart. Table S3. Summary of database annotations.

Supplementary Material 5. Table S5. Differential metabolite information.

Supplementary Material 6.

Supplementary Material 7. Table S2. Differentially expressed gene information.

## Data Availability

The datasets generated and/or analysed during the current study are available in the Genome Sequence Archive (GSA) repository, [CRA039953] (https://ngdc.cncb.ac.cn/gsa/browse/CRA039953).

## Abbreviations

YG: Yellowish-Green
L*: Brightness
a*: Red-green degree
b*: Yellow-blue degree
GA: Gibberellin
ABA: Abscisic acid
ROS: Reactive oxygen species
TEM: Transmission electron microscopy
RHS: Royal Horticultural Society
RHSCC: Royal Horticultural Society Colour Chart
UPLC-MS/MS: Ultra-performance liquid chromatography-tandem mass spectrometry
UPLC-ESI-MS/MS: Ultra-performance liquid chromatography-electrospray ionization tandem mass spectrometry
MRM: Multiple reaction monitoring
RNA: Ribonucleic acid
cDNA: complementary Deoxyribonucleic acid
PC1: Principal component 1
KEGG: Kyoto Encyclopedia of Genes and Genomes
GO: Gene Ontology
Nr: Non-redundant
DEGs: Differentially expressed genes
DAMs: Differentially abundant metabolites
PCR: Polymerase chain reaction
qRT-PCR: quantitative real-time PCR
IPP: Isopentenyl pyrophosphate
DMAPP: Dimethylallyl pyrophosphate
ZEP: Zeaxanthin epoxidase
SGR: Staygreen
NCED1: 9-cis-epoxycarotenoid dioxygenase 1
CCD: Carotenoid cleavage dioxygenase
CCDs: Carotenoid cleavage dioxygenases
DXS: 1-deoxy-D-xylulose-5-phosphate synthase
PSY3: Phytoene synthase 3
GluTR: Glutamyl-tRNA reductase
NYC-like: Non-yellow coloring-like
CAO: Chlorophyllide a oxygenase
F: Forward primer
R: Reverse primer
CM: Cell membrane
CW: Cell wall
Chl: Chloroplast
IC: Granum lamella
M: Mitochondria
N: Nucleus
OG: Osmiophilic globule
RER: Rough endoplasmic reticulum
V: Vacuole
SG: Starch granule
