# Supplementary material for: Integrated transcriptomic and metabolomic analysis suggests a potential regulatory network underlying leaf color transition in Ulmus pumila ‘Jinye’
Source: BMC Plant Biol. 2026 Jun 8;26:1367. doi: 10.1186/s12870-026-09202-5 (PMC13465358; doi:10.1186/s12870-026-09202-5)
Supplement: Supplementary file 1 [file 12870_2026_9202_MOESM1_ESM.docx]

Fig. S1 Transcriptomic profiling of *U. pumila* ‘Jinye’ leaves between the YG vs. Golden stages. (A) Principal component analysis (PCA) of all samples. (B) Volcano plot of differentially expressed genes (DEGs). (C) Numbers of up-regulated, down-regulated, and total DEGs. (D) Gene Ontology (GO) enrichment analysis of DEGs. (E) Top 20 enriched Kyoto Encyclopedia of Genes and Genomes (KEGG) pathways. Data are shown as the mean ± SD of six biological replicates.

Fig. S2 Metabolomic profiling of *U. pumila* ‘Jinye’ leaves between the YG and Golden stages. (A) PCA of metabolite profiles. (B) Volcano plot of DAMs. (C) Pie chart classification of all identified metabolites. (D) Top 20 significantly enriched KEGG pathways. Data are shown as the mean ± SD of six biological replicates.
