# Supplementary material for: Integrated transcriptomic and metabolomic analysis suggests a potential regulatory network underlying leaf color transition in Ulmus pumila ‘Jinye’
Source: BMC Plant Biol. 2026 Jun 8;26:1367. doi: 10.1186/s12870-026-09202-5 (PMC13465358; doi:10.1186/s12870-026-09202-5)
Supplement: Supplementary file 2 [file 12870_2026_9202_MOESM4_ESM.docx]

Table S1. Analysis of leaf color development in *U. pumila* ‘Jinye’ based on the Royal Horticultural Society Large Colour Chart.

| Leaf color stages | Group of color | Number | Name |
| --- | --- | --- | --- |
| Golden | Green -Yellow group | 1A | Brilliant Greenish Yellow |
| YG | Yellow-green group | 144A | Strong Yellow Green |
| Green | Green group | 137B | Moderate Olive Green |

YG: Yellowish-Green; Data are expressed as the mean ± SD (n = 15).

Table S3. Summary of database annotations.

| Annotated Database | Annotated Number | 300≤length<1000 | Length≥1000 |
| --- | --- | --- | --- |
| NR | 28786 (73.96 %) | 8391 (21.56 %) | 20395 (52.40 %) |
| eggNOG | 27304 (70.16 %) | 7372 (18.94%) | 19932 (51.21%) |
| Swissprot | 22276 (57.24%) | 5419 (13.92%) | 16857 (43.31%) |
| KOG | 17310 (44.48%) | 4424 (11.37 %) | 12886 (33.11 %) |
| GO | 19122 (49.13 %) | 4356 (11.19 %) | 14766 (37.94 %) |
| Pfam | 19529 (50.18%) | 3873 (9.95 %) | 15656 (40.23 %) |
| KEGG | 6687 (17.18%) | 1729 (4.44%) | 4958 (12.74%) |

Data are expressed as the mean ± SD (n = 6).

Table S7. Photosynthetic pigment ratios in *U. pumila* ‘Jinye’ in response to exogenous phytohormone treatment.

| Treatment | Total chlorophyll | Carotenoid | Total chlorophyll /Carotrnoid Ratio |
| --- | --- | --- | --- |
| Mock | 1.75 ± 0.16 | 0.45 ± 0.11 | 3.89 |
| GA3+ABA | 0.34 ± 0.09 | 0.57 ± 0.13 | 0.60 |
| GA3 | 0.56 ± 0.11 | 0.48 ± 0.12 | 1.17 |
| ABA | 0.84 ± 0.15 | 0.54 ± 0.11 | 1.56 |

Data are expressed as the mean ± SD (n = 3).

Table S8. Photosynthetic pigment ratios in *U. pumila* ‘Jinye’ in response to exogenous phytohormone treatment.

| Treatment | Total chlorophyll | Carotenoid | Total chlorophyll /Carotrnoid Ratio |
| --- | --- | --- | --- |
| Mock | 0.22±0.01 | 0.56±0.03 | 0.39±0.04 |
| PBZ+ Sodium tungstate | 2.36±0.01 | 0.97±0.01 | 2.55±0.01 |
| Sodium tungstate | 1.74±0.02 | 1.1±0.03 | 1.58±0.02 |
| PBZ | 1.85±0.02 | 1.25±0.02 | 1.48±0.01 |

Data are expressed as the mean ± SD (n = 3).

Table S10. Primers used for RT-qPCR.

| Gene name | Gene ID | Primer sequence 5’ to3’ |
| --- | --- | --- |
| PSY3 | TRINITY_DN22746_c0_g1_i1_1 | CAGTGAGCACCAACGACTAC  TTTGCTACCGCTCTTCCTG |
| CCD | TRINITY_DN17034_c2_g2_i25_5 | CATGAAAACTGGTCTCGCAAC  AGGCTATAGAGGAAGAGGTGG |
| CCD | TRINITY_DN17519_c2_g1_i15_6 | TTAGTTCCTCTGTTCCTCAAGC  CAGCCTTCAACCATCTTTGTG |
| NCED1 | TRINITY_DN22054_c0_g1_i1_3 | CACCCAAAACAATCCCGAAAG  TCTTACCAAAACCCCTTCCAC |
| HEMA | TRINITY_DN15959_c0_g1_i1_2 | TCCCACAATCAAGAAACTCCG  GCTTGCTCACAATTCCCATG |
| NYC1 | TRINITY_DN15911_c0_g1_i7_2 | GCTGTGCAATCCCTAAGAGAAG  ATCCTGCCCCTCTTTAACATC |
| DXS | TRINITY_DN17143_c0_g1_i9_1 | ACCCTCACAAAATCCTTACTGG  TCATATTCACTCTCAGCACGC |
| ZEP | TRINITY_DN15213_c0_g1_i8_5 | GGGCAGTATAGAGGTCCAATTC  AGAGACTCCATCAACCAAACC |

Data are expressed as the mean ± SD (n = 6).
